# Healthy longevity, intrinsic capacity, geroscience and Alzheimer’s disease prevention

**DOI:** 10.1016/j.tjpad.2026.100570

**Published:** 2026-04-22

**Authors:** Bruno Vellas

**Affiliations:** IHU HealthAge Toulouse, France

Alzheimer’s disease remains one of the greatest challenges of aging societies worldwide. Despite major advances in biomarker development and disease-modifying therapies, aging itself remains the strongest and most consistent risk factor for Alzheimer’s disease. Promoting healthy aging likely represents the most powerful and scalable strategy for its prevention.. Geroscience and healthy longevity, supported by the concept of intrinsic capacity, provide a framework that aligns naturally with the prevention-oriented mission of the Journal of Prevention of Alzheimer’s Disease.

**The biological mechanisms that drive aging** and geroscience reframe Alzheimer’s disease as a potentially preventable condition emerging from cumulative, modifiable aging processes [1]. Multiple aging-related biological pathways contribute directly to Alzheimer’s disease risk and progression [2]. Epigenetic alterations, chronic low-grade inflammation, mitochondrial dysfunction, oxidative stress, impaired autophagy and proteostasis, cellular senescence, and immune dysregulation interact with amyloid-β and tau pathology to accelerate neurodegeneration. These mechanisms act synergistically over decades, creating a long preclinical window during which prevention strategies may be most effective. In addition to anti-amyloid agents, the therapeutic pipeline increasingly includes compounds targeting neuroinflammation, microglial dysfunction, metabolic failure, mitochondrial impairment, and cellular senescence. Combination therapies that simultaneously address Alzheimer’s pathology and aging-related mechanisms are particularly promising, as they reflect the multifactorial nature of disease risk. The INSPIRE [3] translational platform developed at the IHU HealthAge illustrates how geroscience can be operationalized for Alzheimer’s prevention. By integrating short-lived experimental models, murine systems, and a deeply phenotyped longitudinal human lifespan cohort, INSPIRE enables the identification of aging biomarkers that precede cognitive decline and Alzheimer’s pathology. Longitudinal data from the INSPIRE-T cohort will explore how biological aging markers—including epigenetic clocks, inflammatory aging signatures, and mitochondrial dysfunction—are closely associated with Alzheimer’s blood biomarkers well before clinical symptoms appear. These studies will probaly reinforce the concept that Alzheimer’s disease develops along a continuum shaped by aging biology.

**The concept of intrinsic capacity**—defined as the composite of an individual’s physical and mental capacities—offers a clinically actionable framework for healthy longevity and prevention across the life course [4]. Declines in intrinsic capacity are strongly associated with frailty, disability, cognitive impairment, and increased Alzheimer’s biomarker burden. Conversely, individuals who maintain higher intrinsic capacity show greater cognitive resilience and healthier brain aging. Vision and hearing correction and maintenance are often omitted from multidomain Alzheimer's disease prevention trials, despite their potential impact. An integrative approach must be integrated in Alzheimer’s prevention, (the “I” from ICOPE: Integrated Care for Older Persons by the WHO). The Intrinsic Capacity composite score and percentiles [5,6] give us the tools to monitor IC and healthy longevity in a manner analogous to monitoring cognition with the CDR score; both cognitive and other functions are needed for a more integrative and long-term prevention strategy. Digital prevention strategies, including platforms such as ICOPE MONITOR, enable large-scale assessment and longitudinal monitoring of intrinsic capacity: vision, hearing, mobility, memory, psychological health and nutrition at a large scale population level to promote healthy longevity [7]. By combining self-assessment tools, telehealth, artificial intelligence, and personalized interventions, these approaches bridge the gap between biological insights and real-world implementation. Such tools align closely with Alzheimer practical, scalable prevention strategies [8].

**The PROSPR (PROactive Solutions for Preserving Resilience)** program [9], developed by the Advanced Research Projects Agency for Health (ARPA-H) highlights the limits of a healthcare model still largely focused on treating established diseases and underline the importance of preserving resilience rather than reacting to decline. PROSPR is grounded in the concept that functional deterioration (Intrinsic Capacity trajectory) begins long before clinical disease becomes apparent. It therefore aims to detect early, subtle changes across physical, cognitive, and physiological domains using continuous, technology-enabled monitoring, including wearable devices and digital biomarkers. This longitudinal approach allows the identification of critical inflection points at which timely intervention may prevent or reverse decline. By combining early detection with personalized, multidomain interventions (e.g., physical activity, nutrition, cognitive engagement, and emerging biological therapies), PROSPR seeks not only to delay deterioration but also to restore functional capacity. PROSPR integrates advanced data analytics and artificial intelligence to enable scalable, population-level deployment. In doing so, it bridges innovation in aging biology with real-world health systems. PROSPR exemplifies a shift from disease-centered medicine to a proactive, function-centered model, with the potential to extend healthy lifespan and preserve autonomy at scale.

Healthy longevity & geroscience align naturally with Alzheimer's disease prevention. By targeting fundamental aging mechanisms, supporting healthy longevity, and advancing rational combination therapies, this framework offers a coherent strategy to delay onset, reduce incidence, and the societal burden of Alzheimer’s disease.

## CRediT authorship contribution statement

**Bruno Vellas:** Writing – original draft.

## Declaration of competing interest

The authors declare that they have no known competing financial interests or personal relationships that could have appeared to influence the work reported in this paper.
